# The nurse′s challenge of caring for patients with chronic obstructive pulmonary disease in primary health care

**DOI:** 10.1002/nop2.135

**Published:** 2018-03-15

**Authors:** Tanja Gustafsson, Lena Nordeman

**Affiliations:** ^1^ Research and Development Center Södra Älvsborg Närhälsan, Research and Development Primary Health Care Region Västra Götaland Borås Sweden; ^2^ Närhälsan Fristad Primary Health Care Center Borås Sweden; ^3^ Faculty of Caring Science, Work Life and Social Welfare University of Borås Borås Sweden; ^4^ Department of Health and Rehabilitation Unit of Physiotherapy Institute of Neuroscience and Physiology Sahlgrenska Academy University of Gothenburg Gothenburg Sweden

**Keywords:** asthma and COPD nursing, chronic obstructive pulmonary disease, nursing, primary health care, qualitative research

## Abstract

**Aim:**

The aim was to describe asthma and chronic obstructive pulmonary disease nurses′ experiences of caring for patients with chronic obstructive pulmonary disease in primary health care.

**Design:**

Descriptive qualitative research.

**Methods:**

Ten asthma and chronic obstructive pulmonary disease specialized nurses were interviewed. Systematic Text Condensation by Malterud was used to analyse the data.

**Results:**

Two main categories were found: the patient‐nurse relationship and available resources. Several challenges emerged when connecting with patients and the nurses found it difficult to individualize care. They struggled with a lack of time and support from other professionals. Being responsible for asthma and chronic obstructive pulmonary disease practice was experienced as positive, but could become overwhelming. The asthma and chronic obstructive pulmonary disease nurses described several challenges and the need for support and resources to provide the best possible care for patients with chronic obstructive pulmonary disease.

## 1 INTRODUCTION

Chronic obstructive pulmonary disease (COPD) is a global problem and a major cause of chronic morbidity and mortality (Global initiative for chronic obstructive lung disease, GOLD 2016). In Sweden, approximately 500,000 people are expected to suffer from COPD and over 2,000 lose their lives every year because of the disease (Swedish Pulmonary Organization 2017). The major cause of COPD is smoking. Other risk factors are heredity, occupational exposure and socioeconomic status. Initially, sufferers may lack symptoms, but will develop later leading to health‐related disorders. Early symptoms are prolonged and repeated episodes of coughing, with or without mucus and wheezing in the chest. In most cases, breathlessness during effort appears later in the course of the disease. Several organs can be affected depending on the disease′s severity (ibid.). Smoking cessation is the single most important measure and leads to prolonged survival along with decreased symptoms. Physical activity reduces problems with dyspnoea and increases the quality of life (SBU 2000).

### 1.1 Background

Guidelines for the care of patients with COPD are developed on an international, national and regional level. The international organization GOLD (2016) provides guidance for COPD management and prevention strategies. The national guidelines in Sweden emphasize the importance of collaboration between professionals in the care of patients with COPD, such as specialized nurses, physicians, physiotherapists, dieticians and psychologists (The National Board of Health and Welfare 2015). Nurse‐led asthma and COPD practices in primary healthcare centres (PHCC) can offer organized care and provide education and support to help patients improve their ability to manage the disease and its effects on their lives. Studies show that PHCCs with asthma and COPD nurse practice (ACNP), experience fewer COPD exacerbations and hospitalizations (Lisspers et al., 2014; Löfdahl et al., 2010). In western Sweden, a regional guideline has been developed from the national guidelines, which contain brief facts and descriptions of treatment (The region Västra Götaland 2013). The focus is on diagnostics and management of patients with COPD at PHCCs.

Reports show a need to structure the care of patients with COPD in primary health care (The National Board of Health and Welfare 2014). A survey performed in western Sweden indicated that 80% of the involved PHCCs had an ACNP (Thorn et al., 2008). An approved ACNP, includes a nurse specialized in asthma and COPD, booked visits, telephone counselling, structured investigations, patient education, support for smoking cessation and follow‐up visits (Kull et al., 2008). The asthma and COPD specialized nurses′ (ACN) experience of patient education can fluctuate between insecurity and security, as presented in a qualitative study by Zakrisson and Hägglund (2010). The nurses wished for more structured patient education, time, collaboration and support from other professions, to enable individualization of care. Structured ACNPs have been shown to be cost‐effective in primary health care (Lindberg, Ahlner, Ekström, Jonsson, & Möller, 2002). Reports indicate that the care of these patients is generally adequate, however, there are several areas that need to be improved (The national Board of Health and Welfare 2014). Despite the knowledge that a structured management can contribute to evidence based, good and safe care of patients with COPD, reports show deficiencies in applying the knowledge, leading to unequal care (Lindberg et al., 2002; The National Board of Health and Welfare 2015).

## 2 AIM

The aim of this study was to describe ACNs′ experiences of caring for patients with COPD in primary health care.

## 3 METHOD

### 3.1 Study design

Qualitative interviews were chosen to describe the ACNs′ experience of caring for patients with COPD. All 201 PHCCs in the western region of Sweden were randomly selected and a total of 70 interest‐in‐participation requests were sent to managers at each PHCC by e‐mail or letter. Half the managers responded and provided the ACNs′ contact information. Eight nurses declined participation and ten ACNs were included. Details concerning participants are provided in Table 1. All participants provided their written informed consent. None had an established relationship with the authors.

**Table 1:** Participants characteristics

Informant	Age	Years as RG^a^	Years as ACN	PHCC size^b^	Hours/week/1000 patients^c^
1	45	15	10	>8000	1.1
2	61	40	11	>8000	1.2
3	58	35	20	>8000	2.0
4	55	34	3	>8000	1.6
5	58	36	14	>8000	1.6
6	40	15	1	<7999	1.6
7	62	38	6	<7999	1.7
8	52	30	8	<7999	1.3
9	52	18	6	<7999	1.8
10	64	40	20	<7999	1.3

a Registered nurse.

b Number of patients listed at the Primary Health Care Center.

c On Asthma and COPD Nurse Practice.

### 3.2 Data collection

Data were collected from December 2015–June 2016. The first author interviewed the ACNs at their PHCC. The taped semi‐structured interviews lasted between 30–50 minutes. Informants were asked to describe their experiences of caring for patients with COPD. In‐depth questions such as: “Can you tell me more about…?” and “What do you mean?” were asked. After the interviews, the first author transcribed the recordings verbatim.

### 3.3 Data analysis

Data were analysed through Systematic Text Condensation (STC) as described by Malterud (2012). The method is a stepwise process suitable for analysis of qualitative data and includes four steps. First, all interviews were read to obtain an overall impression of the material. Preliminary themes were formed. The first step is described as “from chaos to theme”. In the next step “from themes to codes”, units of meaning were identified and systematized, representing different aspects of the ACNs′ experiences. The third step was to condensate the units of meaning, “from code to meaning”. In the last step, the condensates were summarized by generalizing descriptions and concepts, “synthesizing – from condensation to descriptions and concepts”.

### 3.4 Ethical considerations

The Regional Ethical Review Board in Gothenburg approved the study. All participants received written and oral information before entering the study.

## 4 FINDINGS

**Figure 1:**
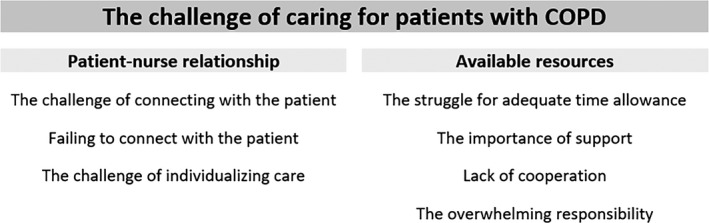
An overview of categories and subcategories

Two main categories regarding the ACNs′ experiences of caring for patients with COPD were generated from the data, the patient‐nurse relationship and available resources. The first category describes the challenges of connecting with the patient and how care was affected when connection failed. The nurses also experienced challenges individualizing care and structuring visits according to patient needs. The second category describes available resources for the nurses at the ACNP and the importance of time, support and cooperation in keeping responsibility from becoming overwhelming. An overview of the findings presented as categories and subcategories are listed in Figure 1.

### 4.1 Category: Patient–nurse relationship

#### 4.1.1 The challenge of connecting with patients

The ACNs in the study found it exciting and challenging to meet patients with COPD. They felt a challenge in approaching patients because of the need for them to adapt to different perceptions of the illness and its treatment. They also felt the need to adapt to the complexity of the disease. The nurses described variation in patients′ receptivity towards information about the disease and its treatment. The nurses experienced that some patients had difficulties perceiving information despite repeated visits, while others understood how to manage their disease and its effects on their daily lives:

“I need to know who is before me […] How can I reach this person? What is their background and educational level? How should I explain so that he understands? What information is needed?” (Informant nr.3)

The ACNs experienced how they played an important role for patients with COPD. By giving advice on physical activity, smoking, medicine, nutrition, vaccination, etc., they had the opportunity to help patients improve their quality of life. Several participants mentioned how they usually attained good contact with patients building the relationship on confidence and trust. The nurses felt that they gained insight into the patient′s situation and easily made contact with them, thus feeling that they created a sense of security for the patient. They also perceived having more time to listen to the patient during the visit than, for example, physicians. The ACNs felt that they provided continuity. Several of the nurses emphasized the importance of reaching the patient and building a good relationship, to help them with smoking cessation, be more physically active and to improve compliance to medication:

“You get very good contact with them and you build close trust, I think. So, you have an important function, I believe.” (Informant nr.9)

#### 4.1.2 Failing to connect with the patient

Several participants experienced a sense of hopelessness and frustration when failing to connect with the patient. One of the nurses described this as the most challenging part of meeting the patient. Failing to connect would result in the visit losing its meaning:

“Some (patients) just come here and do as they are told. They come here, blow (spirometry) and leave. That way, there is no contact with discussion and talk.” (Informant nr.6)

Most participants had trouble helping patients with smoking cessation, while viewing it as an exciting task. They had the opportunity to be involved and rejoiced when a patient managed to stop smoking. There could be a feeling of frustration when patients were not motivated for smoke cessation and it often felt difficult to get them sufficiently motivated:

“Then there are those who are completely introverted, they are not many, but some… you just can′t reach them: then I don′t know what to do!” (Informant nr.6)

#### 4.1.3 The challenge of individualizing care

In our material, several participants shared the view that it was both stimulating and personally developing to work independently with patients on the ACNP. Patient visits involved activities such as spirometry, symptom evaluation, smoking cessation support, inhalation technique and advice on physical activity and nutrition. Visits had more or less the same structure but the nurses tried to adapt meetings based on patient needs and disease severity. Visits were also affected by how well nurses knew the patients. One of the nurses described it as easier to motivate patients with less severe COPD, to improve quality of life through various activities. Patients with severe COPD and more negatively affected by the disease made greater demands on the nurse, who felt the need for a more careful approach. Some nurses explained the importance of structure to ensure time for each aspect of the visit:

“I have a number of things I check off to make sure that I have everything covered.” (Informant nr.9)

One participant described how she tried to get the patient to focus on the purpose of the visit by talking only about problems related to breathing. Other subjects were avoided:

“When we meet (patients) I maintain strict control of what I must do, because otherwise it takes too long. So, we try to stick to breathing and breathlessness.” (Informant nr.7)

### 4.2 Category: Available resources

#### 4.2.1 The struggle for adequate time allowance

The ACNs described how their way of working depended on several factors; training, experiences, education and guidelines. The physicians and available resources on the PHCC also affect methods. Several nurses mentioned different guidelines and recommendations they used in the care of patients with COPD. Participants described how they tried to adjust their practice to current guidelines as much as possible. This created structure, but the guidelines were perceived as extensive and difficult to apprehend:

“The guidelines from the National Board of Health and Welfare are completely new. I have read them and… they will really make strong demands on many of us asthma and COPD nurses.” (Informant nr.1)

Several nurses experienced a lack of further education. The pharmaceutical industry often accounted for further education, often evenings. A few expressed searching for new knowledge and research as important and interesting, yet time consuming and difficult to perform in the time available at the ACNP:

“I think my problem is that it is difficult, during stressful working days, keeping up to date, taking in all the new knowledge.” (Informant nr.10)

Most participants experienced too little time for asthma and COPD patients. Some mentioned recommendations regarding the number of hours per week and listed patients. However, this is not mandatory at the PHCCs and therefore not often followed:

“Someone has calculated it… not something you need to follow. It is more like you need to have an asthma and COPD nurse at least a few hours weekly at a PHCC.” (Informant nr.1)

The nurses felt that they had freedom and control of their ACNP, but the lack of time resulted in stress over obligations. Several participants expressed their inability to meet the patients as often as wanted, due to prioritizations, which could result in some follow‐ups by telephone. Some nurses often handed over the responsibility to maintain contact with the patients in case of increased impairment. One of the nurses expressed the importance of taking time, showing interest despite lack of time.

#### 4.2.2 The importance of support

Participants expressed the importance of cooperation with other professions in the care of patients with COPD. However, experiences of cooperation varied among nurses. Some experienced well‐functioning collaboration with physicians. When the nurses felt an interest in this group of patients among the physicians, it was easier to discuss patient‐related issues and get help with eventual problems. This support was very valuable:

“We have excellent cooperation with the doctors; they are very competent, I think, regarding patients with COPD, inhalation and so on. They do their part, are competent and you can talk to them. “(Informant nr.6)

#### 4.2.3 Lack of cooperation

Several of the nurses expressed vulnerability when cooperation with physicians was inadequate. Some described increased responsibility for asthma and COPD patients, in the absence of an attending physician. The care of these patients was often experienced as difficult and complex and responsibility felt overwhelming. One participant felt that the physicians at her unit had appointed her as expert, but she felt insecure in that role. Several of the nurses had similar experiences; they felt the physicians relied too heavily on them. The nurses missed working together with the physicians and according to some, the lack of cooperation was caused by fluctuating interest and knowledge of COPD. In addition, lack of time had a negative impact on their cooperation:

“It can feel burdensome at times, constantly shouldering all that responsibility alone.” (Informant nr.5)

Some participants enjoyed good cooperation with the physio‐ and occupational therapists at the rehab clinics. It was considered a plus when contact with the rehabilitation unit was functioning well and they saw them as an important resource, especially in the care of patients with severe COPD:

“We have a physiotherapist we can refer to that has more training and is a little more familiar with our group of patients.” (Informant nr.2)

Despite the fact that nurses thought support important, several experienced a lack of cooperation with the rehab clinic. They described having no contact with physiotherapists or occupational therapists: instead patients were recommended to contact optional rehab clinics themselves. They also missed feedback when patients had visited a rehab clinic:

“They (the patients) get a brochure and a telephone number to primary care rehabilitation. Then the patients can go to any physiotherapist they want.” (Informant nr.5)

Some nurses had the opportunity to contact a dietician if needed, while the remaining participants did not consult them. The nurses could help patients get nutritional drinks, but felt they lacked the necessary knowledge in that area:

“Calculating calories and such, I can′t do that. It's more like ′take some fruit compote and heavy cream, eat small portions and then add some nutritional drinks.” (Informant nr.8)

Most of the nurses did not consult counsellors or psychologists in the care of patients with COPD.

#### 4.2.4 The overwhelming responsibility

The nurses in the study described having considerable responsibility for the ACNP, which felt mostly positive. Some told of how they were responsible for coordinating care based on the patients’ needs. They contacted physicians, rehab clinics and others when needed. They felt that this role facilitated for both the patient and PHCC. However, this responsibility could feel overwhelming, especially regarding patients with the most severe COPD. In these cases, the support of pulmonary specialists was important. Some of the nurses experienced successful cooperation with pulmonary care clinics:

“I have some (patients) with severe COPD, I have one on oxygen, with an attending physician. I meet him and confer and she is also in touch with the pulmonary care clinic. So, I feel I get help.” (Informant nr.9)

Several of the nurses, however, experienced a lack of cooperation with specialized care. The nurses felt that they lacked sufficient knowledge and resources to take care of patients with the most severe COPD. They felt frustrated when they could not give these patients the best possible care due to lack of support from pulmonary specialists:

“How can you get them to feel better, when their spirometry curve is so small that it is barely visible? How can you make them feel better? It feels hopeless. It feels like there is nothing more to do.” (Informant nr.6)

To meet severely ill patients could affect the nurses emotionally, especially when they felt a lack of support from the physicians at the PHCC and pulmonary care unit:

“Sometimes I feel like I have nothing more to give, these (patients) are so ill. Maybe there is nothing more I can do. Sometimes it feels burdensome, because they are so sick, I think. They barely have the strength to walk from the waiting room to my room. (Informant nr.3)

In some PHCCs, the nurses were solely responsible for the ACNP, while in others there were two ACNs. Several were stressed by not being able to share responsibility. They were worried about what would happen in case of an eventual leave of absence. They missed discussing matters with a colleague with knowledge of the subject. To have an ACN colleague created a sense of security.

## 5 DISCUSSION

COPD is a complex disease making great demands on primary health care. Our study indicates that caring for patients with COPD is a challenge to the ACN. There are barriers to overcome for both nurses and patients. To help and guide the patient towards improved health and disease management, the nurse must connect with the patient and form a good relationship. It is important for nurses to have sufficient resources and support from other professionals to keep responsibility from becoming overwhelming.

### 5.1 Patient–nurse relationship

The ACNs in the study described the challenges when caring for patients with COPD due to the complexity of the disease and differences in how patients experienced its effects on their lives. Connecting with patients can be difficult, leading to frustration. Studies show that patients want improved care and there is a lack of self‐management support and understanding of their condition (Wortz et al., 2012). Patients felt confused, frustrated and wanted to learn more about their illness. They also experienced a lack of information about the disease and medication. Ekman et al. (2011) imply that person‐centred care improves health outcomes and patient satisfaction through interaction between patients, families and healthcare professionals. Seeing the patient as a partner, listening to their perception of their condition and experiences and documenting the patient narrative and health plan are keys to person‐centred care.

Participants in our study had diverse backgrounds and experiences of this group of patients, which is yet another factor having an impact on care. Some nurses focused more on practical aspects, for example spirometry and tried to check off several mandatory measures during the visit, while others tried to focus and adjust the visit to individual needs. A previous study by Lundh, Rosenhall, and Törnkvist (2006) also shows this division: nurses were either task‐ or individual‐oriented, depending on their experience, knowledge and access to support. Despite the fact that most of the nurses in our study desired individualized care, they struggled with balancing the mandatory parts of the visit with patient focus. An explorative observational study, from 2009, shows similar findings (Österlund Efraimsson, Klang, Larsson, Ehrenberg, & Bjöörn, 2009). The nurses had, in general, an adequate structure for the patient visit and tried to focus on patient histories. They gave information to the patients about self‐management and smoking cessation, but the use of motivational dialogue was limited. Care was rarely summarized in a written treatment plan.

### 5.2 Available recourses

In our study, participants described the resources at their disposal in the care of patients with COPD. Several wished that they had had more time to provide as good care as possible. Some of the nurses described feeling forced to follow “a manuscript” during visits to allow time to gather required information. By focusing mainly on patients′ breathing some participants felt they could provide adequate structure to the visits. The lack of time also prevented them from seeing the patients as often as they considered necessary and made it difficult to follow guidelines to the desired extent. An important factor in providing good quality management in the care of patients with asthma and COPD is the amount of time reserved for ACNP (Carlfjord & Lindberg, 2008).

It was mainly through education supported by the pharmaceutical industry that participants could partake of new research and guidelines. Some described having no designated time to partake in research or involve themselves in current subjects and studies. Their time was filled with patient visits. In a survey, Perez, Wisnivesky, Lurslurchachai, Kleinman, and Kronish (2012) detected barriers to adherence to COPD guidelines. The results showed that primary care providers (physicians) had little familiarity with recommendations, low self‐efficacy and time constraints, which had negative effects on adherence.

Cooperation with other professionals differed between ACNs in the study. Only a few participants worked in inter‐professional teams. Most considered it especially important to collaborate with physicians and physiotherapists, but several witnessed a lack of cooperation, particularly the nurses working alone as an ACN at the unit and missed discussing problems with colleagues. Thorn et al. (2008) demonstrates, in a study in Sweden 2008, that larger PHCCs had better opportunities to design the care of this group of patients based on guidelines and recommendations. Several participants in our study expressed how they wished physicians had better knowledge of the care of patients with COPD and felt a lack of support, especially for patients with the most severe form of COPD. The nurses described enjoying responsibility, but only to a certain degree. They believed the physicians trusted the ACNs′ competence, occasionally more than the nurses wished for, according to some. The division of responsibility was somewhat unclear, as described by some nurses and they expressed how they would have preferred that physicians would take more medical responsibility. Harris, Hayter, and Allender (2008) describes in a study the factors affecting nurses′ and physicians′ cooperation. Similar to our study, the nurses experienced a lack of teamwork and wished for more involvement by physicians in the care of patients with COPD. They also felt they had to act as “substitute doctors” and held too much medical responsibility for the patient instead of focusing on nursing. The physicians, on the other hand, described how they were only involved in acute conditions; the remaining care was delegated to specialized nurses, which made them feel less skilled. Some considered the nurses more competent, having greater knowledge of this group of patients. The nurses also shared this opinion.

The ACNs in our study described caring for patients with advanced COPD as the most difficult part. The responsibility felt overwhelming, due to a lack of support from physicians and pulmonary specialists. They also felt shortcomings in their skills and competence. In Zakrisson and Hägglund (2010) study participants described the importance of support. The nurses with support from colleagues and management felt more secure regarding patient education. When support was inadequate, it resulted in insecurity and had negative effects on care of patients with COPD. Another qualitative study (Kentischer, Kleinknecht‐Dolf, Spirig, Frei, & Huber, 2017) from an emergency care hospital reported that nurses experienced ambivalence regarding complex nursing care situations. Perceiving situations as positive challenges or overwhelming burdens depended on the contextual conditions and the nurse′s personality. Gardiner et al. (2010) describes potential barriers regarding resistance to communicating with the patient with advanced COPD. The uncertain illness trajectory and uncertainty among health professionals of how patients would perceive discussions about the condition and prognosis were described as barriers.

Several of the ACNs wished for better cooperation with rehab clinics, considering the importance of physical activity in patients with COPD at all stages. The nurses in our study described how they usually discussed the importance of physical activity during visits, giving patients advice and support in this matter. Sometimes patients were recommended contacting a rehabilitation clinic. The eventual visits were not often followed up. National guidelines in Sweden (The National Board of Health and Welfare 2015) emphasize the importance of assessing physical capacity and offering patients with COPD support regarding physical activity. When assessing physical activity, the use of the six‐minute walk test is recommended and should be offered to patients in a stable phase and also to identify patients with an increased risk of mortality and hospital admission (ibid.). Much is involved in visits, which jeopardizes the time nurses had to thoroughly discuss physical activity and measures to decrease breathlessness and other symptoms. Cooperation with physiotherapists as experts in this area should allow ACNs more time to focus on the patients′ other important needs and wishes.

### 5.3 Strengths and weaknesses of the study

Qualitative methods are suitable to the study of human experiences. A strength of our study was that the interviews provided a deeper understanding about the nurses′ lived experiences of caring for patients with COPD and the factors effecting care. The first author had previous experience of in‐depth interview. The informants had a range of work experiences and worked in different sized PHCCs located in different areas, which gave variability to the findings. Ten ACNs participated, which was the original plan and was considered a sufficient amount according to current conditions. Fewer participants would have resulted in too little data.

A weakness of the study was the loss of participants due to stressful working conditions and perhaps in some cases, insecurity about their competence. The loss of participants less confident in their roles as asthma and COPD specialized nurses may have had effects on the study, with the result that the findings cannot be generalized. However, it is possible that the findings can be transferred and provide increased understanding of similarly complex nursing situations.

The first author is an experienced ACN and that could affect the interviews according to her preconceptions. Being aware of the phenomenon and asking the participant to develop thoughts regarding feelings and reflections about the subject could lead to the avoidance of negative effects due to preconceptions. Over time, the first author became more secure and aware of her preconceptions and adjusted her technique.

The data was analyzed by Systematic text condensation and was considered suitable for this study. Malterud (2012) describes how the method offers a stepwise process of intersubjectivity, reflexivity and feasibility and maintains methodological quality.

### 5.4 Implications

When implementing new guidelines and recommendations and when improving existing methods, it is crucial to consider factors effecting care situations. The findings in our study could be used to understand differences regarding management of the care of patients with COPD. From these nurses’ experiences, parallels can be drawn to other nurse‐led clinics, such as diabetes and cardiovascular. The complexity of caring for patients with COPD needs to be explored from both the patients’ and nurses′ point of view, as well as their interaction. To improve and make care more equal, there is a need for greater research in how to decrease the effects of barriers between patients and nurses and how to improve necessary support to nurses.

## 6 CONCLUSION

Caring for patients with COPD is a challenge to the ACN. There are barriers to overcome for both the nurse and patient. To help and guide the patient towards improved health and management of the disease, the nurse must connect with the patient and develop a good relationship, requiring sufficient resources and support from other professionals to avoid the responsibility from becoming overwhelming.

## ETHICAL APPROVAL

The Regional Ethical Review Board in Gothenburg approved the study, Dnr: 524‐15.

## ACKNOWLEDGEMENTS

We thank all interviewees for their participation. Financial support was obtained from the research and development council of Södra Älvsborg, Region Västra Götaland, Sweden. A scholarship was received from Astma‐ Allergi‐ och KOLsjuksköterskeföreningen (ASTA).

## CONFLICT OF INTEREST

The authors report no conflict of interest.

## AUTHOR CONTRIBUTIONS

TG and LN was responsible for the study conception, design, and drafting of the manuscript. TG performed the data collection and both authors performed data analysis. LN made critical revisions to the paper and supervised the study. Both authors read and approved the final manuscript.
